# Fish Consumption and Blood Mercury Levels

**DOI:** 10.1289/ehp.1307997

**Published:** 2014-05-01

**Authors:** Edward Groth

**Affiliations:** Groth Consulting Services, Pelham, NY

The article “Dietary Predictors of Maternal Prenatal Blood Mercury Levels in the ALSPAC Birth Cohort Study” (Golding et al. 2013) is seriously flawed.

The paper’s central conclusion is that there was no strong correlation between fish consumption and blood mercury levels in their study population. The authors suggest that several other dietary items should be investigated as potentially significant sources of mercury exposure.

Possible additional sources of mercury in the diet may be worth exploring, but the fact that fish consumption is the primary driver of elevated methylmercury in the body has been established empirically beyond any reasonable doubt by numerous studies, such as the NHANES (National Health and Nutrition Examination Survey) (Mahaffey et al. 2009). This relationship has also been quantified in a pharmacodynamic model developed by the U.S. Environmental Protection Agency (Rice et al. 2003). The data used to build that model came from clinical studies with volunteers who ate measured amounts of fish with known mercury content.

Most populations in which the blood mercury–fish consumption relationship has been studied have included a cohort with a comparatively high level of fish intake and significantly elevated blood mercury (i.e., > 10 µg/L). For example, in the NHANES surveys, about 7–9% of subjects exceeded 5 µg/L, and high values were > 30 µg/L. Also, in the United States, most popular seafood items are very low in mercury, but some exceptions, especially tuna, have a substantial market share, so a significant subset of Americans often eat higher-mercury fish.

In contrast, the population that Golding et al. studied appears to have had quite low overall blood mercury levels. From their Figure 1, about 98% had mercury levels < 5 µg/L, and their maximum value was 12.8 µg/L. With such low overall mercury levels and such a narrow distribution, it is not surprising that no dietary factors correlated strongly with the (minimal) variation in blood mercury. In addition, the authors’ data on fish consumption are generic (white fish, oily fish, shellfish). Different fish species vary by > 100-fold in mercury content. Without detailed data on fish species eaten, the mercury doses of individual subjects cannot be estimated. The result, imprecise exposure classification, would also tend to reduce any association between (generic) seafood intake and blood mercury.

In short, although Golding et al. observed no association between fish intake and blood mercury in a British cohort with generally low blood mercury levels, such an association has been repeatedly demonstrated in U.S. studies, in populations that include people with higher blood mercury. That subset with elevated blood mercury includes people at risk for nontrivial adverse health effects (e.g., Freire et al. 2010; Lederman et al. 2008; Masley et al. 2012; Oken et al. 2008; Sagiv et al. 2012). Because mercury cannot be kept out of fish, and the nutritional benefits of fish consumption are substantial, the most appropriate public health response to this risk is to advise high-end fish consumers to choose primarily low-mercury fish.

A paper that suggests that there is a minimal relationship between fish consumption and blood mercury—and fails to explain the somewhat limited conditions under which that may be so or, more importantly, to address the extensive literature showing conditions under which it is not so—has the potential to seriously confuse an already very complex public debate over fish consumption advice. It is disappointing, to say the least, that the authors did not address these issues.
